# The Foreign Journals: Medical Statistics

**Published:** 1842-04

**Authors:** 


### MEDICAL STATISTICS.

*Results of Revaccination in the Prussian Army in the year* 1840. By Dr. LOHMEYER.

IN the last year there were vaccinated in all the regiments together, 43,522 individuals. On these the cicatrices from previous vaccinations were

| | |
|---|---|
| distinct in . . . | 34573 |
| indistinct in . . . | 6177 |
| not discernible in . | 2772 |

The pustules produced by the present vaccinations were

| | |
|---|---|
| regular in their course in . . | 20952 |
| and irregular in . . . . . | 8820 |
| and no effect at all was produced in | 13750 |

The vaccinations that had thus taken no effect were repeated

| | |
|---|---|
| with success in . . | 2831 |
| without success in . | 8958 |

The numbers of genuine pustules produced were as follows:

| | | |
|---|---|---|
| 1 to 5 | in | 10021 |
| 6 to 10 | in | 5875 |
| 11 to 20 | in | 4171 |
| 21 to 30 | in | 885 |

Of those who had been successfully vaccinated in 1840 and previously there were attacked during the past year with varicella . . 7
with varioloid . . 2
with true variola . 1

In the past year more frequently than in any other, the vaccination was performed with lymph taken from genuine and regular vaccine pustules on adult persons; for past experience has consequently led military practitioners to be more strongly convinced that for the vaccination of adults lymph taken from adults is to be preferred to that taken from children, and that it produces a more powerful reaction and more strongly-developed pustules. And that the pustules thus produced do actually protect from the contagion of smallpox is proved by this, that in the 4th corps, in which the plan has been long followed of vaccinating adults with lymph taken from good pustules in other adults, there has not occurred during three years a single case of any variety of smallpox among those in whom the vaccine pustule ran its ordinary course, nor more than one case among those who were unsuccessfully revaccinated, although smallpox was constantly more or less rife in the neighbourhood in which the corps was quartered.

The results of past years have shown a regularly increasing ratio in the proportion of successful vaccinations to the number altogether vaccinated; and this increase has continued in the year 1840, for of 43,522 revaccinated, 20,952 have exhibited genuine and regular pustules. Of all the revaccinations, therefore, 48 per cent. were successful; while in 1839 the proportion was only 46, in 1838 and 1837 only 45, in 1836, 43, in 1835, 39, in 1834, 37, and in 1833 only 31 per cent. The proportions varied in 1840 between 40 per cent. and 60 per cent. in different regiments.

With respect to the advantage of revaccination as a preventive of smallpox, the experience of 1840 proves it in a striking manner; for during that year there occurred only 74 cases in the whole army, and of these 46 were cases of varicella, 21 of varioloid disease, and only 7 of genuine smallpox. Of the last 2 proved fatal; and neither of the patients had been revaccinated after his enlistment. In general, just as in former years, most of the cases of spurious and genuine smallpox occurred in recruits soon after their enlistment and before they had been revaccinated. Of those who had been revaccinated it has been already stated that only 10 were affected, and of these 7 had varicella, 2 varioloid disease, and only 1 genuine variola.

*Medicinische Zeitung. April* 21, 1841.

---

*Results of the Revaccination in the Hanoverian Army in the Years* 1837-8-9. From the Army Medical Reports. By Dr. MÜHRY.

SINCE the year 1837 revaccination has been practised in the Hanoverian army, and to such an extent as to include not only those who bore unsatisfactory cicatrices of former vaccinations or none at all, but all without distinction, and then annually all the recruits. The results, with respect to the susceptibility of the influence of the vaccine matter, were as follows:

In 1837 there were revaccinated, 4610, of whom 351 bore no cicatrices of the former vaccinations.

Of these the vaccination was completely successful in . . . 506
incompletely in . . . . . 916
without influence in . . . . 3188

In 1838 there were revaccinated, 2699, of whom 187 bore no cicatrices.

Of these the vaccination was completely successful in . . . 268
incompletely in . . . . . 661
without influence in . . . . 1770

In 1839 there were revaccinated 2057, of whom 143 had no cicatrices.

And of these the vaccination was completely successful in . . . 294
incompletely in . . . . 578
without influence in . . . . 1175

The whole results, therefore, were as follows, that revaccination in 9366 persons, of whom 681 had no cicatrices, was successful in 1068, or in 11 per cent.; and partially successful in 2155, or in 27 per cent.

The persons revaccinated were for the most part between twenty and twenty-one years old, except in 1837, when about half of them were more or less further advanced in age. The number of punctures was from eight to sixteen. The matter was chiefly taken from the children* of the married soldiers, and conveyed direct from arm to arm; and when these sources of supply were insufficient, lymph was obtained from children in private practice, or dry lymph was used, or the lymph from those who had been successfully revaccinated. The success was considered complete when on the seventh day there was a pustule with a central depression, clear lymph, and an areola. Between that and the mere healing of the puncture there were a variety of intermediate forms, of which papillæ with red margins were reckoned among the cases with no result, and pustules which had crusts formed on the seventh day were reckoned among the incompletely successful cases. The revaccinated were not excused from service unless they were feverish, but that rarely occurred.

On a comparison of these results with those obtained in the Prussian army, and in Wurtemberg, both in the army and among civilians during an epidemic of variola, Dr. Mühry points out that the proportion of perfectly successful cases varied in some measure. In the Hanoverian army it amounted to more than $\frac{1}{10}$, in the Prussian army it was about $\frac{1}{2}$; in Wurtemberg, in the army, about $\frac{1}{3}$, among civilians nearly $\frac{1}{2}$. The proportion of imperfectly successful cases on the contrary was greatest in the Hanoverian army. In it, it was more than $\frac{1}{4}$, in the Prussian army $\frac{1}{6}$, in the Wurtemberg army less than $\frac{1}{4}$, and in the people $\frac{1}{5}$. Taking however the completely and incompletely successful cases together, the results were nearly the same in all, the proportions being in the order followed above, $\frac{1}{3}$, $\frac{3}{5}$, $\frac{3}{5}$ and rather more than $\frac{3}{5}$.

Among the confirmations which this report affords of the conclusions arrived at by Professor Heim (Historisch-kritische Darstellung der Pockenseuche) are the following: The cicatrices of former vaccinations afford neither by their number nor by their characters any indication of what will be the result of revaccination. A repetition of revaccination after previous failures not unfrequently produces a complete or incomplete result. The lymph of good pustules after revaccination is in no degree inferior for the purpose of revaccination to the lymph from children. In those who have had variola the susceptibility of revaccination exists in no less a degree than in those who have been vaccinated; for of 112 pitted with smallpox, 16 received complete revaccination, 21 incomplete, and 75 none at all, that is, in nearly the same proportion as among the vaccinated. On the contrary, it appeared that after varioloid disease scarcely any were susceptible of revaccination; for of 34 such who were revaccinated, one only showed any result, and that was imperfect, affording an additional proof that variolois is an actually varioloid disease, a revariola.

*Hannoversche Annalen.* Heft ii. 1840.

---

### *Statistics of Suicide in France.*

The following are the numbers of suicides throughout the whole of France during the last four years, but the numbers are not exactly correct, as many doubtful cases of suicide are ranged, according to the wishes of friends, among accidental deaths:

| | |
|---|---|
| 1836 | 2,310 |
| 1837 | 2,413 |
| 1838 | 2,556 |
| 1839 | 2,717 |

The number has thus been increasing annually.

*Gazette Médicale de Paris. Aout* 7, 1841.

* Compare on this subject the remarks in the Report of the Results of Revaccination in the Prussian Army, in which adults were vaccinated with matter from other adults.

